# VNA-Based Vector Reflection Coefficient Measurement Technique for Powered RF Signal Generators

**DOI:** 10.3390/s26113590

**Published:** 2026-06-05

**Authors:** Emre Cetin, Aliye Kartal Doğan, Anil Cetinkaya, Erkan Danaci

**Affiliations:** 1 Spark Measurement, 06530 Ankara, Türkiye; emre_cetin@sparkmeasure.com; 2 TÜBİTAK UME, 41470 Kocaeli, Türkiye; aliye.dogan@tubitak.gov.tr (A.K.D.); anil.cetinkaya@tubitak.gov.tr (A.C.); 3 Department of Computer Engineering, Faculty of Engineering and Natural Sciences, Iskenderun Technical University, 31200 Hatay, Türkiye

**Keywords:** RF signal generator, vector reflection coefficient, VNA, impedance matching, measurement method

## Abstract

The reflection coefficient measurement of the RF signal generator output is clear when the signal generator output is turned off, as no interfering signal is present. However, measuring the reflection coefficient while the signal generator output is turned on creates complexity, as the generator’s output power can interfere with the reflected signal. A vector network analyzer (VNA) is the reference instrument for measuring the reflection coefficient, capturing both the magnitude and phase of scattering parameters. For measuring the active output of a signal generator, the signals created by the generator and the VNA must be isolated to prevent signal mixing and interference. This paper proposes a unique method to measure the output reflection coefficient of an RF signal generator when the output is on, using a VNA configured for one port reflection coefficient measurement. The method involves tuning the VNA receiver to a frequency slightly offset to the generator’s output. Simultaneously, selecting a narrow intermediate frequency bandwidth (IFBW) reduces the receiver’s noise floor and also eliminates out-of-band interference. As a result, the VNA and the generator operate in different frequency bands to avoid interferences between them, enabling accurate magnitude and phase measurements. To automate the process, a Windows-based software has been developed. This software automates the measurement sequence, controls generator power levels and VNA sweep parameters, captures both the magnitude and phase of the reflection coefficient, and records the result data. It also supports measurement at different output power levels, enabling characterization across a wide range of operating conditions.

## 1. Introduction

**Figure 1:**
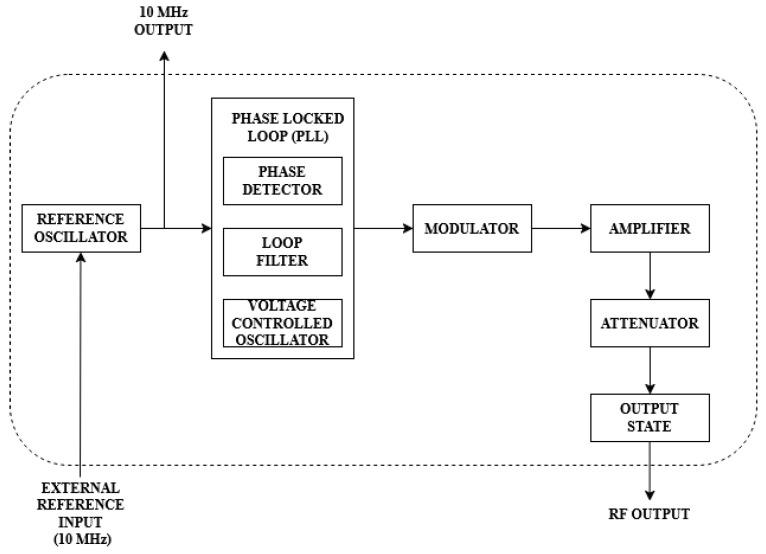
Typical RF signal generator block diagram.

A radio frequency (RF) signal generator is an electronic instrument designed to generate periodic or arbitrary waveforms for the purpose of testing and characterizing electronic equipment. A typical RF signal generator consists of several critical subsystems, primarily a phase-locked loop (PLL)-based frequency synthesizer and an output stage, which commonly includes an amplifier and a variable attenuator [1]. The basic block diagram of the signal generator is shown in Figure 1.

In signal generators, the reference oscillator is typically implemented using a crystal oscillator (XO) or an oven-controlled crystal oscillator (OCXO). OCXO can provide superior frequency stability and reduced sensitivity to temperature variations. Although the reference output is often 10 MHz, other values may be employed depending on the signal generator architecture.

In RF signal generator topology, the reference frequency is sent to a frequency synthesizer, which is commonly based on a PLL block. A PLL comprises a phase/frequency detector, loop filter, and a voltage-controlled oscillator (VCO) arranged in a negative-feedback configuration [2]. The synthesizer output is phase-locked to the reference, so any phase or frequency deviation produces an error signal that is filtered and applied to the VCO control input. Within the PLL, frequency dividers and multipliers permit the generation of a wide range of output frequencies from the fixed reference by adjusting the division factor N. When PLL achieves lock, the VCO output frequency is equal to N×(reference frequency). This structure is commonly referred to as a Fraction-N PLL, enabling fine frequency resolution and a broad tuning range [2].

The signal at the PLL output is a continuous-wave (CW) signal. A modulator block, which may be optional depending on the signal generator type, can be included to apply modulation formats such as amplitude modulation (AM), frequency modulation (FM), phase modulation (PM), or pulse modulation on the CW signal. Further optional blocks include amplifiers and attenuators in the signal generator, as shown in Figure 1 within a dashed line. These stages are used to set and control the output power level, generally from +20 dBm down to −130 dBm. Step attenuators are commonly employed for precise attenuation control, and amplifier chains may consist of multiple paths according to the required frequency range and power level. All of these mentioned subsystems contribute to the output reflection coefficient of the signal generator. However, the amplifier and attenuator blocks are generally dominant as they consist of multiple signal paths and impedance transformations, which affect the output impedance match.

The output impedance of an RF signal generator is nominally 50 Ω, which corresponds to an ideal impedance under perfect matching conditions [3]. However, in practice, the actual output impedance deviates from this 50 Ω, leading to the reflection coefficient values that can vary significantly from −∞ dB to 0 dB. These variations are due to internal components, such as amplifiers, attenuators, and switches, that affect the overall output impedance. In addition, many signal generators incorporate mechanical output switches or internal isolation mechanisms that further modify the impedance seen at the connector. As a result, these factors contribute to the non-ideal behaviour of the generator’s output impedance.

Measuring the output vectorial reflection coefficient of an RF signal generator is simple when the generator is powered off. In this case, a VNA, which has been the main reference instrument for vectorial reflection coefficient measurements since the 1960s, determines the generator’s output reflection coefficient accurately by performing a one-port calibration at the reference plane which is the signal generator output port [4]. However, the complications can appear when the RF signal generator is powered on. The signals applied by the generator and the signal injected by the VNA to measure the reflection coefficient interfere at the VNA’s receiver, thus preventing accurate reflection coefficient measurements. Additionally, if the generator’s output power is sufficiently high, it can damage the VNA’s receiver [5]. Many laboratories do not measure the output reflection coefficient of signal generators because of the potential risk of damaging the VNA.

Historically, laboratories have relied on standing wave ratio (SWR) values provided by instrument manufacturers to estimate uncertainties in RF power measurements [6]. However, since SWR is a scalar quantity that reflects only the magnitude of the impedance mismatch and does not capture phase information, it typically represents a worst-case estimate rather than a precise characterization of mismatch effects. Consequently, the uncertainty contribution coming from the impedance mismatches has traditionally been significant in power measurement [7,8]. VNA-based calibration methods are widely adopted in RF metrology owing to their superior measurement capability; however, accurate characterization of the reflection coefficients involved in the measurement chain is essential, since these parameters may vary depending on connector conditions, internal signal paths, and operating states of the instruments [9]. This limitation is particularly relevant in RF power sensor calibration, where source mismatch must be characterized accurately to transfer traceability reliably [10].

In this study, we propose a novel method for measuring vectorial reflection coefficient of an RF signal generator while it is powered on. This approach is inspired by the “Hot $S_{22}$ Measurement Technique” used to characterize power amplifiers, in which two internal VNA sources are offset in frequency to prevent signal mixing during the reflection coefficient measurements [11]. Similar active and hot-parameter measurement approaches have also recently been applied for nonlinear microwave and millimeter-wave device characterization using VNA-based setups operating under large-signal conditions [12]. In addition, frequency-separated active measurement configurations have previously been investigated in nonlinear microwave characterization systems employing source-pull and load-pull techniques [13]. By applying a similar frequency offset strategy, the reflected signal can be isolated from the generator output, enabling accurate measurement even when the generator is active. To facilitate practical applicability, we have also developed a Windows-based software to automate this measurement procedure.

## 2. Vector Reflection Coefficient Measurement Method Using VNA While RF Signal Generator Is Powered On

**Figure 2:**
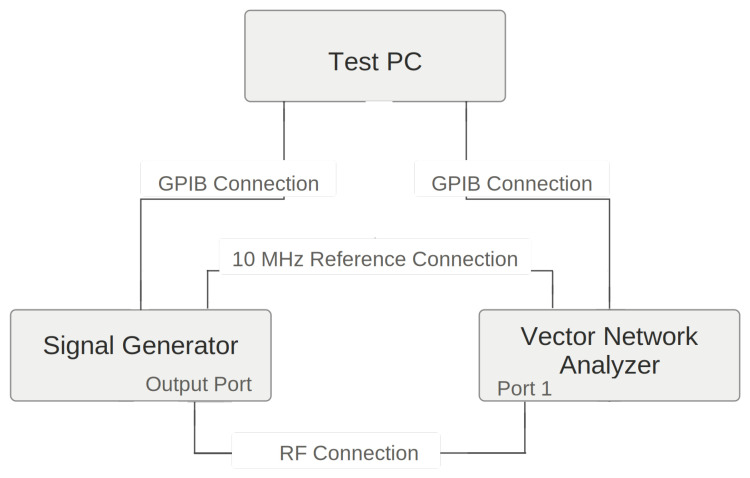
The block diagram of the vector reflection coefficient of RF signal generator measurement setup.

The method proposed in this study uses a frequency-offset technique, in which the operating frequencies of the signal generator and the VNA are set with a slight offset, thereby preventing signal mixing at the VNA receiver. Basic frequency-offset measurement technique is explained in a Keysigth application note [12,14]. A VNA can have many IFBW modes which are specified in its options. Simultaneously, a narrow IFBW is chosen, ensuring that the VNA’s receiver processes only the measurement tone from the VNA, not the tone generated by the signal generator. Selecting a narrow IFBW is also critical when the signal generator’s phase noise is non-ideal as phase noise-induced sidebands of the generator’s tone may otherwise leak into the VNA’s receive band and degrade measurement accuracy. Finally, synchronization of the internal frequency references of both the signal generator and the VNA is recommended to eliminate frequency offsets arising from mismatched reference sources. The diagram of the proposed measurement setup is shown in Figure 2.

A test PC is used to control the signal generator and VNA, as shown in Figure 2. The output of the RF signal generator is directly connected to the VNA measurement port to measure the reflection coefficient of the RF signal generator. Reference synchronization between the RF signal generator and the VNA is carried out by connecting the reference 10 MHz inputs and outputs.

**Figure 3:**
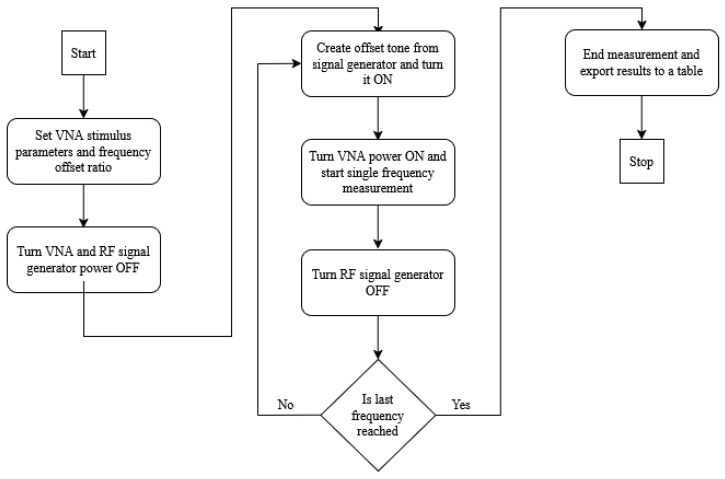
Flowchart illustrating the vector reflection coefficient of RF signal generator measurement method.

The flowchart illustrating the proposed reflection coefficient measurement method in this study is shown in Figure 3.

The proposed measurement method is initiated by setting the stimulus parameters of the VNA and the frequency offset ratio. The VNA and the RF signal generator are initially powered off. An offset tone is subsequently generated using the signal generator and activated prior to measurement. Then, the VNA is powered on, and a single-frequency measurement is performed at the selected frequency point. Following completion of the measurement, the RF signal generator output is powered off. The procedure then verifies whether the final frequency in the predefined sweep has been reached. If additional frequency points remain to be measured, the other offset tone is regenerated, and the measurement sequence is repeated for the next frequency. Upon completion of measurements across all frequency points, the process is terminated, and the acquired data are exported into a table for subsequent analysis.

**Figure 4:**
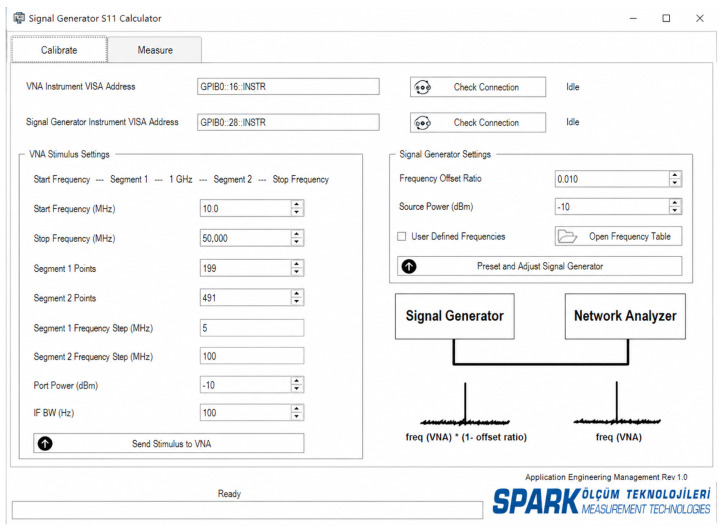
User interface of the developed application for vector reflection coefficient of RF signal generator measurement.

A Windows-based software has been developed to carry out the operations outlined in this flowchart. The graphical user interface of the developed software is shown in Figure 4.

The application offers configurable settings, beginning with the entry of the VISA resource addresses for the VNA and the RF signal generator. Clicking the “Check Connection” button checks the connection status. If the instruments are properly connected, the adjacent status label (originally displaying “Idle”) turns to a green “Connected” label. If the connection has not been established, this label turns to a red “No Instrument Connected” label. Furthermore, if a connection exists but the device is not supported, the status changes to an “Unsupported Instrument” label.

**Figure 5:**
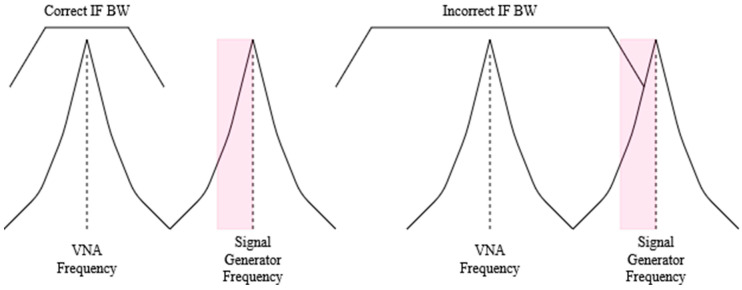
Effect of chosen IFBW for non-ideal phase noise.

The user configures the VNA stimulus settings by enabling a segmented frequency sweep to enhance frequency coverage. The sweep is divided into two segments: the first segment spans from the user-specified start frequency to 1 GHz, and the second segment extends from 1 GHz to the user-defined stop frequency. The user must also specify the number of frequency points for each segment. The application dynamically displays the frequency step for both segments as the user adjusts these values. Finally, the measurement IFBW must be configured. Although 100 Hz is recommended as a default, an IFBW as narrow as 10 Hz can be used when the RF signal generator exhibits elevated phase noise which comes at the expense of sweep time. The importance of selecting an appropriate IFBW is illustrated in Figure 5, where the effects of non-ideal phase noise from both the VNA source and the signal generator can significantly influence the measurement results. Narrower IFBWs help reduce received noise and improve dynamic range by filtering out noise outside the passband. On the other hand, the received signal at the VNA receiver will be higher if improper IFBW is chosen, which can damage the VNA’s receiver.

Upon completing the configuration steps, the user should click the “Send Stimulus to VNA” button, prompting the software to apply the specified settings to the VNA. Subsequently, a manual one-port calibration has to be performed to eliminate deterministic errors arising from measurement cables, adapters, and the VNA’s internal components.

For the RF signal generator, two parameters which are the source power and the frequency offset ratio are particularly critical. The ability to adjust the source power provides insight into how the output reflection coefficient varies with the RF signal generator’s power level. The frequency offset ratio $\left(f_{OR}\right)$ defines the spacing between the VNA’s stimulus frequency $\left(f_{VNA}\right)$ and the signal generator’s frequency $\left(f_{SG}\right)$ according to Equation (1).

(1) $$f_{\mathrm{SG}}=f_{\mathrm{VNA}}\cdot \left(1-f_{\mathrm{OR}}\right)$$

The application allows frequency offset ratio values in the range of 0.001 to 0.1, with a default setting of 0.01. Lower offset ratios provide a closer proximity between the VNA and signal generator frequencies. However, this requires a narrower IFBW, especially in the presence of excessive phase noise from the RF generator. The optimal configuration of these parameters depends on the characteristics of the RF signal generator under the test. Upon completion of all configuration steps, the user should click the “Preset and Adjust Signal Generator” button, and the software will apply the specified frequency and power settings to the RF signal generator via VISA communication. This action not only presets the instrument to the desired operational state but also initiates the internal frequency synthesis and establishes the output power level.

Additionally, the software supports a user-defined frequency list. If this option is disabled, the VNA will measure only the fixed frequency points defined in the sweep segments. When the “User Defined Frequencies” checkbox is selected, the user can specify the frequencies to be measured via the “Open Frequency Table” button. In this case, care must be taken to ensure that the segment definitions include these user-selected frequencies to avoid interpolation artifacts by the VNA during measurement.

**Figure 6:**
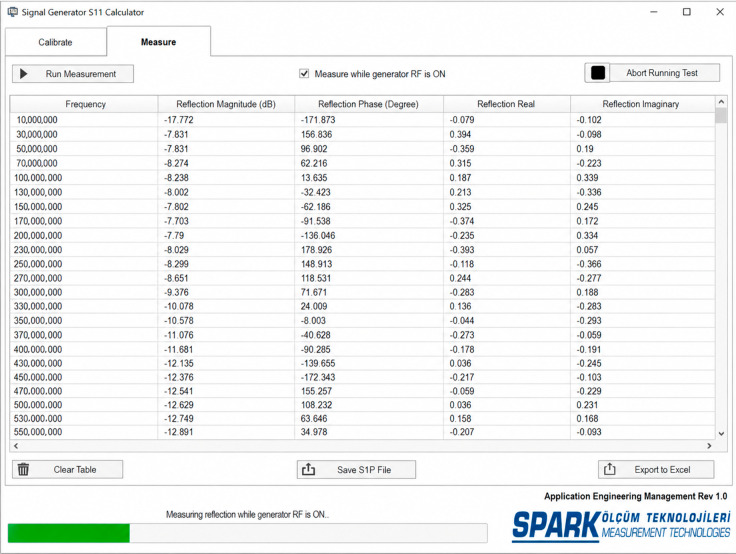
Live measurement display in the developed software.

After completing configuration steps, the user can initiate the measurement by selecting the “Run Measurement” button located in the “Measure” tab. A screenshot illustrating this interface is shown in Figure 6.

During measurement execution, a 50 ms delay is introduced after setting the signal generator frequency to allow the frequency to settle before the VNA captures data. Settling time refers to the period required for the output of a signal generator to stabilize within an acceptable error band after a frequency change. Following this delay, the application captures the magnitude and phase of the reflection, as well as the real and imaginary components of the reflection coefficient, continuously updating and displaying the results in a real-time results table to facilitate dynamic monitoring. In addition, the user interface includes a progress bar that indicates measurement status throughout the acquisition process.

The user may abort an ongoing measurement at any time by clicking the “Abort Running Test” button. Additional functions include controls to clear the results table, save the data as an S1P file (one-port reflection coefficient), and export the results to Microsoft Excel. A checkbox labelled “Measure while generator RF is ON” enables users to perform comparative measurements with the RF signal generator in both powered-on and powered-off cases.

## 3. Experimental Results of the Proposed Method

In the experiments, two different signal generators, E8257D manufactured by Keysight and SMA100B manufactured by Rohde & Schwarz up to GHz, were used as Devices Under Test (DUTs). The Keysight E8257D supports a user-adjustable internal step attenuator, available via Option 1E1, allowing manual control over attenuation settings. In contrast, the R&S SMA100B does not provide a manual attenuator adjustment for the user; attenuation is managed internally without direct user intervention. Using the developed software, a series of measurements was conducted under various power levels and attenuation settings to characterize the reflection coefficient under different operational conditions.

Two VNA models were employed to measure the reflection coefficient of the RF signal generators under test. A E5080A model VNA manufactured by Keysight was used for measurements up to 1 GHz, whereas a N5225A PNA Microwave Network Analyzer manufactured by Keysight was used for measurements up to 40 GHz. The E5080A model VNA was selected for lower-frequency measurements because high-frequency analyzers like the N5225A model VNA can exhibit reduced dynamic range at low frequencies, particularly due to internal coupler roll-off outside the optimum operating band.

To determine the error terms of the VNA, 85056A model mechanical calibration kit with a sliding load was utilized, and port 1 was designated as the measurement port for characterizing the output of the RF signal generators, although the developed application supports the use of alternative ports as measurement reference planes. Calibration corrects for systematic errors introduced by test cables, adapters, and the instrument’s internal test set, thereby improving the accuracy of the measured reflection coefficient.

For frequency stepping, two frequency segments were defined. Segment-1 covers the range from 1 MHz to 1 GHz, while Segment-2 spans from 1 GHz to 40 GHz. During calibration, a 1 MHz frequency increment was selected for Segment-1, and a 500 MHz frequency increment was selected for Segment-2 to balance measurement resolution and sweep time.

An IFBW of 100 Hz was used for all measurements, and single-frequency points were acquired using the VNA’s continuous-wave (CW) sweep feature. At each frequency point, 101 samples were recorded and averaged to derive a representative value, thereby reducing random noise contributions. The VNA source power was set to −10 dBm for the reflection coefficient measurements. The RF signal generators were then configured at different output power levels to evaluate the influence of varying attenuation and power on the measured reflection coefficient.

The datasheets for the Keysight E8257D and R&S SMA100B model signal generators specify the standing wave ratio (SWR) as the metric for reflection characteristics [6,15]. However, these specifications do not clarify whether the reported SWR applies to conditions with the RF output powered on or powered off. The measured reflection coefficient results in this work were compared with the stated SWR values, as SWR is the available manufacturer-provided metric for characterizing reflection performance.

**Figure 7:**
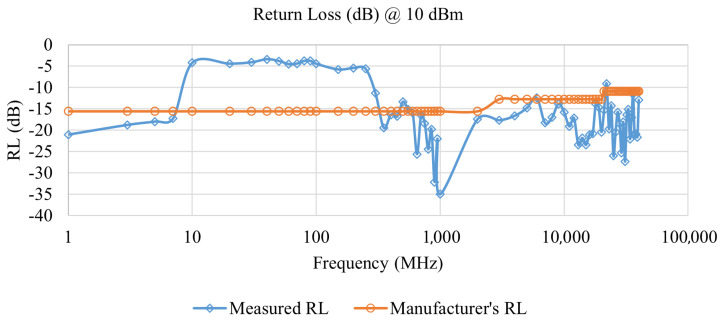
Keysight E8257D RL measurement at +10 dBm power level (0 dB attenuator).

For Keysight E8257D, measurement results performed at +10 dBm output power with the internal attenuator set to 0 dB are given in Figure 7. Measured return loss (RL) represents the RL measured by the developed software, whereas the manufacturer RL represents the manufacturer’s RL derived from SWR values given in the datasheets of the RF signal generators.

As shown in Figure 7, RL deteriorates at low frequencies when the signal generator is operated at high output power levels. This behavior is expected because instruments designed for wide frequency coverage often exhibit degraded performance at the edges of their specified range, particularly at lower frequencies where internal components such as couplers and amplifiers are optimized for high-frequency operation. Additionally, operating with zero attenuation at high power levels does not improve impedance matching, resulting in poorer reflection characteristics and, consequently, higher measured RLs.

**Figure 8:**
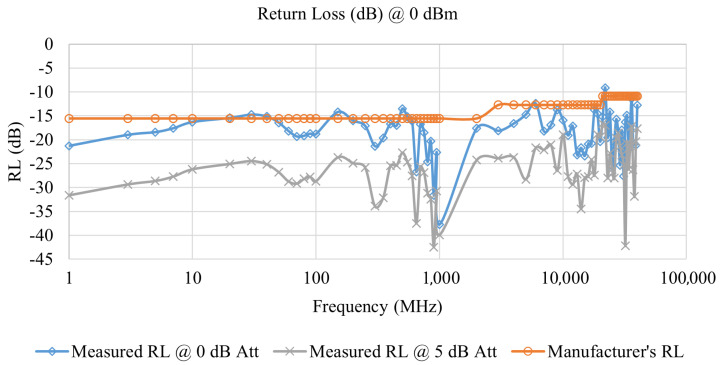
Keysight E8257D RL measurement @ 0 dBm power level 0 dB and 5 dB attenuation.

For Keysight E8257D model signal generator, measurements were performed at 0 dBm output power with the internal attenuator set to 0 dB and 5 dB, respectively, as shown in Figure 8.

As shown in Figure 8, the RL at 0 dBm with 0 dB attenuation exhibits similar behaviour above 1 GHz due to the use of the same attenuator setting when compared to the +10 dBm case. However, at lower frequencies the RL improves, which can be attributed to the internal amplifier architecture of the E8257D signal generator. A comparison with the results obtained using a 5 dB internal source attenuator was performed for the E8257D, showing further improvement in the measured RL. This enhancement is consistent with the expected effect of source attenuation on impedance matching, leading to measured values that fall within the RL specifications.

These observations indicate that, depending on the internal architecture of the signal generator, the RL is significantly influenced by both output power level and attenuator setting. In particular, higher internal source attenuation generally improves reflection characteristics by reducing impedance mismatch at the output of the RF signal generator.

**Figure 9:**
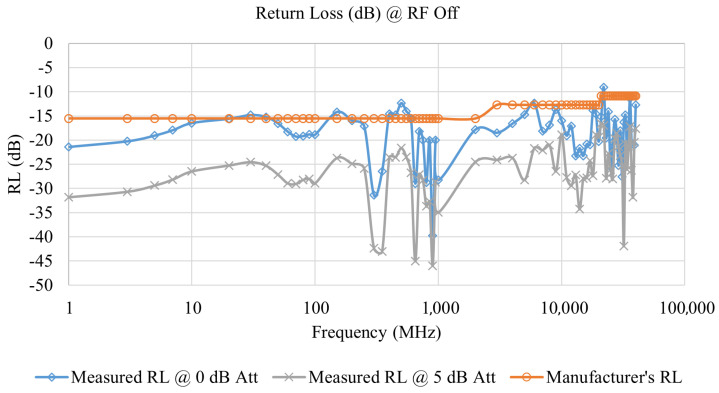
Keysight E8257D RL measurement when RF power is off @ 0 dB and 5 dB attenuation.

Also, further measurements were conducted using the Keysight E8257D signal generator with the RF output powered off. The RL measurements at both 0 dB and 5 dB source attenuation settings, together with the manufacturer-specified RL limits, are presented in Figure 9 for comparison.

As shown in Figure 9, turning off the RF output does not significantly affect the RL measurements of the Keysight E8257D model signal generator. This behavior can be explained by the internal architecture of the E8257D. Even when the RF output is disabled, the generator’s internal circuitry remains directly connected to the RF output port, and no RF switch isolates the internal circuits from the output when the RF is turned off. Instead, the instrument simply disables the RF signal generation while the output port continues to present the internal network and impedance characteristics to the measurement setup. Therefore, the measured RL is dominated by the internal source attenuator and associated front-end network rather than by a physical output isolation. In contrast, other signal generators, such as the R&S SMA100B, can utilize mechanical output switches that isolate the output from internal circuitry when the RF is powered off, resulting in substantially different reflection characteristics under the same conditions.

**Figure 10:**
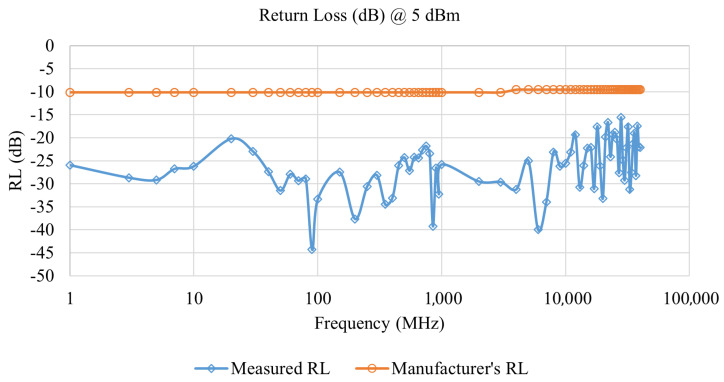
R&S SMA100B RL measurement at 5 dBm power level.

**Figure 11:**
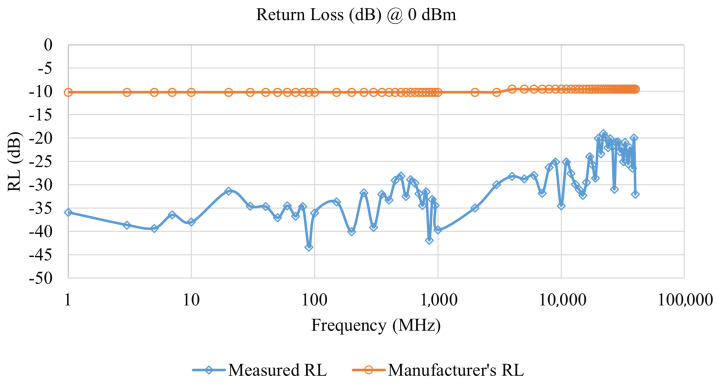
R&S SMA100B RL measurement at 0 dBm power level.

Similar tests were performed using the R&S SMA100B model signal generator [15]. The particular SMA100B unit used in these experiments does not support a high-level output power option across its full frequency range. Therefore, RL measurements were conducted at +5 dBm and 0 dBm output power levels. SMA100B model signal generator does not provide manual external attenuation control via its front panel or remote interface. Therefore, a 0 dB attenuation setting was effectively used for the +5 dBm case, while the R&S firmware automatically applied approximately 10 dB of internal source attenuation for the 0 dBm case to achieve the requested output level. Measurement results for these two cases are shown in Figure 10 and Figure 11.

**Figure 12:**
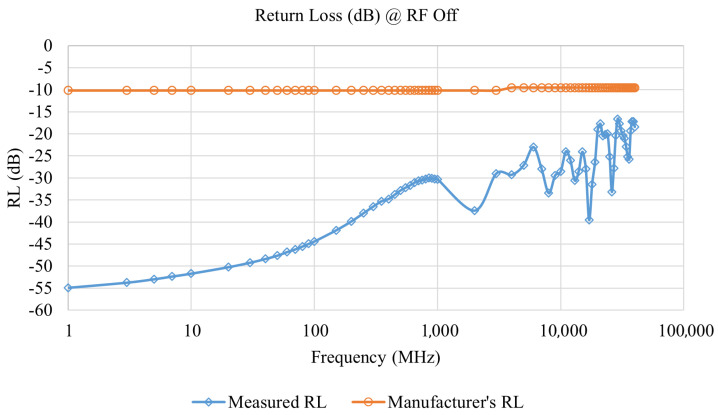
R&S SMA100B RL measurement at 0 dBm power level when RF is powered off.

As noted previously, the R&S SMA100B implements an internal output network that can differ in behavior when the RF output is disabled. This architecture includes signal path switching that effectively isolates the RF output from active circuitry when the RF is turned off. As a result, the RL measurements obtained with the RF output disabled can be quite different when compared to the RF output enabled. This behavior is shown in Figure 12. Only a single power level result is presented there because measurements at all examined power levels yielded similar outcomes due to the signal generator’s internal switching mechanism.

## 4. Discussion and Conclusions

The method proposed in this study enables accurate measurement of the reflection coefficient of RF signal generators using VNAs, which do not have a nonlinear parameters measurement option. Until now, the SWR values provided by manufacturers when RF signal sources were shelved were accepted as the output port reflection coefficient specification for RF signal sources. This study explains how to measure the vectorial reflection coefficient of an RF signal source’s output port while applying power to the VNA, without damaging the VNA, and thus enables the addition of characteristics to RF signal sources than the manufacturer’s specifications. RF signal generators are widely used in RF power meter calibrations and absolute power measurements in which an accurate representation of source mismatch is crucial. Traditionally, signal generator manufacturers’ datasheets only specify SWR to characterize the output impedance matching. SWR reflects only the magnitude of the impedance mismatch and is typically indicated as a worst-case value within a specified frequency band. Therefore, using SWR values to estimate mismatch often leads to higher uncertainty estimates and less accurate impedance mismatch characterization. Previous studies on RF power measurement uncertainty evaluation have also shown that when only the magnitude of the reflection coefficient is available, mismatch uncertainty must be estimated using simplified assumptions and non-Gaussian distributions, whereas vectorial reflection coefficient information enables a more accurate mismatch characterization and uncertainty evaluation [16]. In this study, a complete RF power measurement uncertainty budget was not evaluated, since the primary contribution of the proposed method is the direct vectorial measurement of the reflection coefficient of an active RF signal generator. Nevertheless, accurate vectorial characterization of the reflection coefficient is expected to improve impedance mismatch estimation in RF power measurements and consequently reduce the associated measurement uncertainty.

The method proposed in this study enables direct measurement of the vector reflection coefficient, including both magnitude and phase, of an RF signal generator while it is actively generating a signal. By capturing the complete complex reflection behaviour at the generator output, this approach can reduce the contribution of source mismatch to the overall uncertainty in absolute power measurements and improve the fidelity of uncertainty analysis compared to traditional SWR-based approaches.

To validate the proposed vector reflection coefficient measurement method in this study, SWR values provided by RF signal source manufacturers were compared with SWR values obtained from newly measured vector reflection coefficients. With the widespread adoption of this method, the changes in vector reflection coefficient values measured after periodic calibrations of RF signal sources will be comparable. Future scientific studies are recommended to focus on comparing vector reflection coefficient values measured over many years or on comparing the vector reflection coefficients obtained using the method proposed in this study with new vector reflection coefficient measurements resulting from improvements to this method. For further studies, it is recommended that a detailed uncertainty analysis of the proposed reflection coefficient measurement method be performed, with additional studies aimed at improving the overall measurement uncertainty.

## Data Availability

No new data were created or analyzed in this study.
